# TRIM21 chimeric protein as a new molecular tool for multispecies IgG detection

**DOI:** 10.1186/s43141-022-00396-3

**Published:** 2022-07-28

**Authors:** Anelize Felicio Ramos, Leonardo Antônio Fernandes, Franciane Batista, Bianca de Souza Vieira, Mayerson Thompson, Jacó Joaquim Mattos, Maria Risoleta Freire Marques, Maria de Lourdes Borba Magalhães, Gustavo Felippe da Silva

**Affiliations:** 1 Biochemistry Laboratory, Center of Agroveterinary Sciences, State University of Santa Catarina, Lages, Santa Catarina 88520-000 Brazil; 2 Research and Development Department, Bioclin®, Belo Horizonte, MG 31.565-130 Brazil; 3 Biochemistry Laboratory, Federal University of Santa Catarina, Florianopolis, Santa Catarina Brazil

**Keywords:** Antibody, Protein A, Protein G, Immunoglobulin, Protein engineering, ELISA

## Abstract

**Background:**

The production of monoclonal antibodies for immunoglobulin detection is not cost-effective, while polyclonal antibody production depends on laboratory animals, raising concerns on animal welfare. The widespread use of immunoglobulins in the pharmaceutical industry and the increasing number and variety of new antibodies entering the market require new detection and purification strategies. The Tripartite motif-containing protein 21 is a soluble intracellular immunoglobulin G receptor that binds to the constant region of immunoglobulin G from various species with high affinity. We hypothesized that using this protein as an antibody-binding module to create immunoglobulin detection probes will improve the portfolio of antibody affinity ligands for diagnostic or therapeutic purposes.

**Results:**

We created a chimeric protein containing a mutated form of the C-terminal domain of mouse Tripartite motif-containing protein 21 linked to streptavidin to detect immunoglobulin G from various species of mammals. The protein is produced by heterologous expression and consists of an improved molecular tool, expanding the portfolio of antibody-affinity ligands for immunoassays. We also demonstrate that this affinity ligand may be used for purification purposes since imidazole elution of antibodies can be achieved instead of acidic elution conditions of current antibody purification methods.

**Conclusion:**

Data reported here provides an additional and superior alternative to the use of secondary antibodies, expanding the portfolio of antibodies affinity ligands for detection and purification purposes.

**Supplementary Information:**

The online version contains supplementary material available at 10.1186/s43141-022-00396-3.

## Background

G-type immunoglobulins (IgGs) are widely used as therapeutic agents and molecular tools for antigen detection in immunoassays. However, polyclonal secondary antibody production using laboratory animals has not changed significantly over the last decades, which raises concerns regarding animal welfare. Therefore, the European Union (EU) directive on protecting animals used for scientific purposes urges animal-free alternatives for antibody (Ab)-based technologies. Furthermore, the use of polyclonal secondary Abs presents several limitations, including batch-to-batch variability and size limitations, as well as high cross-reactivity. In addition, after their production, these Abs are chemically coupled to signaling molecules, yielding randomly cross-linked products, which require additional purification procedures resulting in diminished yields and increased production costs [4]. Such limitations impose the search for superior alternatives. Besides the technologies mentioned above, IgGs can be detected/purified by the use of bacterial proteins such as G (from *Streptococcus spp*), A (from *Staphylococcus aureus*), or L protein (from *Peptostreptococcus magnus*). Chimeric proteins using the bacterial proteins mentioned above linked to signal emitting proteins produced several IgG detection probes [14, 18, 20, 21]. The production of A, G, or L also presents disadvantages, given their limited IgG affinities against species or IgG subclasses. For example, Protein A binds rabbit, pig, dog, and cats IgGs, among others, but fails to bind horse, rat, or sheep IgGs [23]. L protein binds only kappa light chains Abs [13], and therefore, alpha light chain Abs are not detected using L protein.

Although protein G possesses a better binding capacity for a broader range of mouse and human IgG subclasses [17], it has an albumin-binding site, limiting its use. Affinity chromatography employing immobilized bacterial proteins for industrial-scale monoclonal antibody purification is costly and accounts for a significant proportion of IgG production costs (60–80%) [5]. Furthermore, IgG purification using Protein A/G coupled resins requires acidic buffers for proper elution, resulting in Ab denaturation and loss of function.

Accordingly, there is a need to develop affinity ligands for IgG detection and purification to decrease costs and increase efficiency. New molecular tools have been created in recent years to expand the pool of antibody binding ligands and overcome the drawbacks associated with bacterial proteins.

A recent paper described camelid nanobodies to replace secondary antibodies [15]. These molecules are expressed in *Escherichia coli*, but present specificity against sole rabbit and mouse IgGs and need further coupling to signal emitting molecules.

The Tripartite motif-containing protein 21 (TRIM21) protein is a soluble intracellular IgG receptor that binds to the Fc region with high affinity [8, 10, 11, 16, 25]. This protein presents a broad antibody specificity, which includes binding to murine, dog, non-human primate, and human IgGs [8] as well as IgM and IgA [9]. As opposed to protein A, TRIM21 binds all human IgG isotypes with high affinity, including IgG 1, 2, 3, and 4 [8]. TRIM21 structure consists of an N-terminal RING domain, a B-box domain, a central coiled-coil domain, and a C-terminal PRYSPRY domain [6]. Earlier studies demonstrated that the portion responsible for IgG binding corresponds to its C-terminal PRYSPRY domain [16].

This study assessed the potential of using the C-terminal domain of TRIM21 PRYSPRY domain as a multispecies IgG binding module to produce chimeric proteins that can detect multi-specie IgGs in immunoassays. We created a chimeric protein containing an N-terminal streptavidin coding sequence followed by a rigid linker composed of four repeats of EAAAK residues and mouse PRYSPRY domain (S370L). The streptavidin portion will allow the coupling of up to four biotinylated molecules per tetramer, including other biotinylated molecules such as enzymes and fluorophores. Data reported here introduces a new and improved molecular tool for IgG detection comparable to the well-established bacterial A and G proteins, expanding the possibilities for biotechnological developments in immunoassays.

## Results

### Protein engineering, expression, and purification

**Fig. 1:**
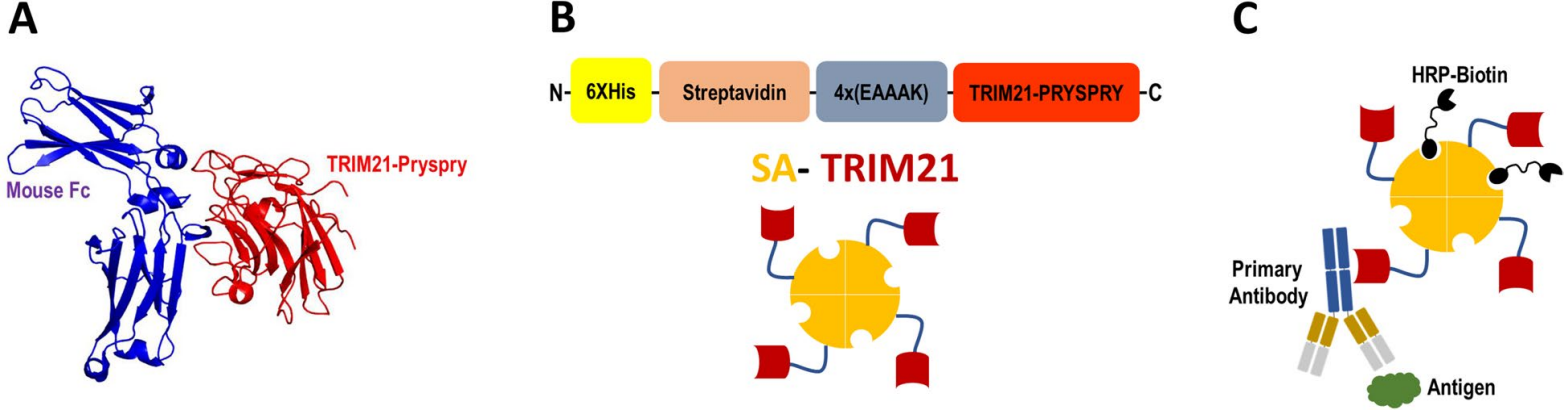
Schematic diagram showing chimeric protein. **A** A 3D-structure of Mouse IgG (blue) bound to the PRYSPRY domain of wild-type mouse TRIM21 (red). **B** The primary and quaternary structure of SA-TRIM21. In red is represented the PRYSPRY domain of TRIM21. Purple represented the rigid EAAAK linker, orange shows the streptavidin domain and yellow the Poli-histidine tag. **C** The graphical summary of SA-TRIM21 recognizing the antigen/antibody complex and performing signaling by biotin-HRP capture

The three-dimensional structure of the C-terminal PRYSPRY domain of mouse TRIM21 (Red) in complex with mouse IgG (Blue) is represented in Fig. 1A, demonstrating that binding occurs between TRIM21 and the Fc domain of the immunoglobulin. Furthermore, competition binding experiments reported earlier showed that TRIM21 binds to the Protein A and G binding site in the Fc domain of IgGs [6]. A schematic diagram of the chimeric protein is shown in Fig. 1B. The N-terminal end of the protein encloses a poly-histidine tag for purification purposes followed by the streptavidin core gene as described by Cantor [19], a rigid [EAAAK]_4_ peptide repeat, and, lastly, the C-terminal PRYSPRY domain of mouse TRIM21 (S370L mutant).

SA-TRIM21 chimeric protein was expressed as inclusion bodies followed by denaturation using Guanidine Hydrochloride and protein refolding through dialysis. Denaturation and refolding are essential, assuring endogenous biotin is removed, and all streptavidin binding sites are unoccupied. Glucose and glycine present in dialysis buffer assisted streptavidin refolding in previous studies [12]. Therefore, these compounds were used to help chimeric protein refolding. Streptavidin is toxic for many *E.coli* strains, and alternative strains are used for streptavidin expression. Here, we used *E.coli* BL21(DE3)pLysS to circumvent toxicity, and free biotin (2 mg/L) was added as earlier reported [7]. A total of 10 mg of soluble purified chimeric protein was obtained per liter of culture. Purified protein was stored at −20 ^°^C and used for immunoassays as described below.

### IgG and animal serum detection using SA-TRIM21

**Fig. 2:**
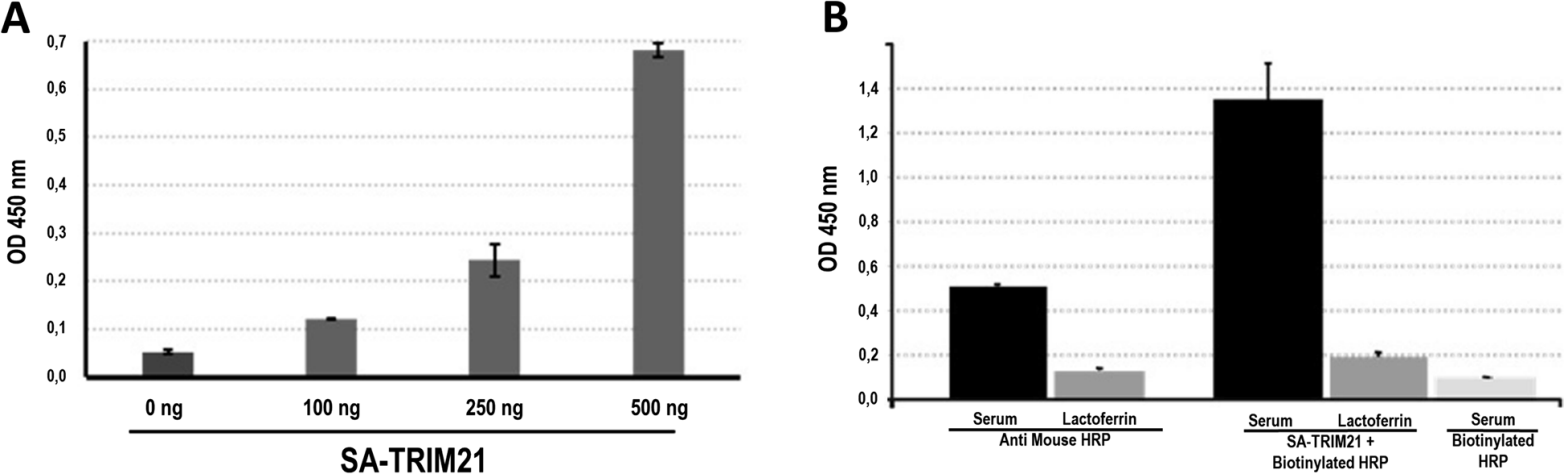
SA-TRIM21 functional assays. **A** The performance of streptavidin domain: SA-TRIM21 (0–500 ng) was coated into polystyrene plates followed by detection using Biotin-HRP (1:2500). **B** Detection of immobilized mouse serum. Mouse serum (50 ng) was coated onto polystyrene plates and detected using HRP-coupled anti-mouse IgG (Sigma, 1:10.000) or 500 ng SA-TRIM21 followed by incubation with biotinylated HRP (dil:1:2500). Control experiments were performed using mouse IgG and biotinylated HRP in the absence of SA-TRIM21. Experiments were performed in triplicates, and error bars represent standard deviation. **A**, **B** Wells were washed with PBST, 50 μL chromogenic substrate TMB was added to the wells, incubated for 10 min, followed by the addition of 50 μL H_2_SO_4_ to quench the reaction followed by OD_450_ nm reading using an ELISA plate reader

We first investigated the performance of the streptavidin domain by SA-TRIM21 immobilization into microplates followed by incubation with biotin-HRP. Figure 2A shows that the streptavidin domain is functional, capturing biotinylated HRP efficiently in a dose-dependent manner. Next, we investigated SA-TRIM21 ability to bind mouse serum on a direct ELISA assay. Figure 2B shows that biotin-HRP coupled SA-TRIM21 (0.5 μg/well) detected mouse serum (50 ng) more efficiently than commercial polyclonal anti-mouse IgG coupled to HRP. Control experiments were performed by immobilizing human lactoferrin, which produced a negligible signal, confirming the specific binding. These data demonstrate that the chimeric protein retains both domains’ functionality.

We investigated the best stoichiometric ratios between SA-TRIM21 and biotinylated HRP (Fig. S1). The SA-TRIM21:HRP ratio that produced the highest signal/noise was 4:1, suggesting that additional HRP may cause sterical hindrance that causes PRYPSRY domain loss of function.

**Fig. 3:**
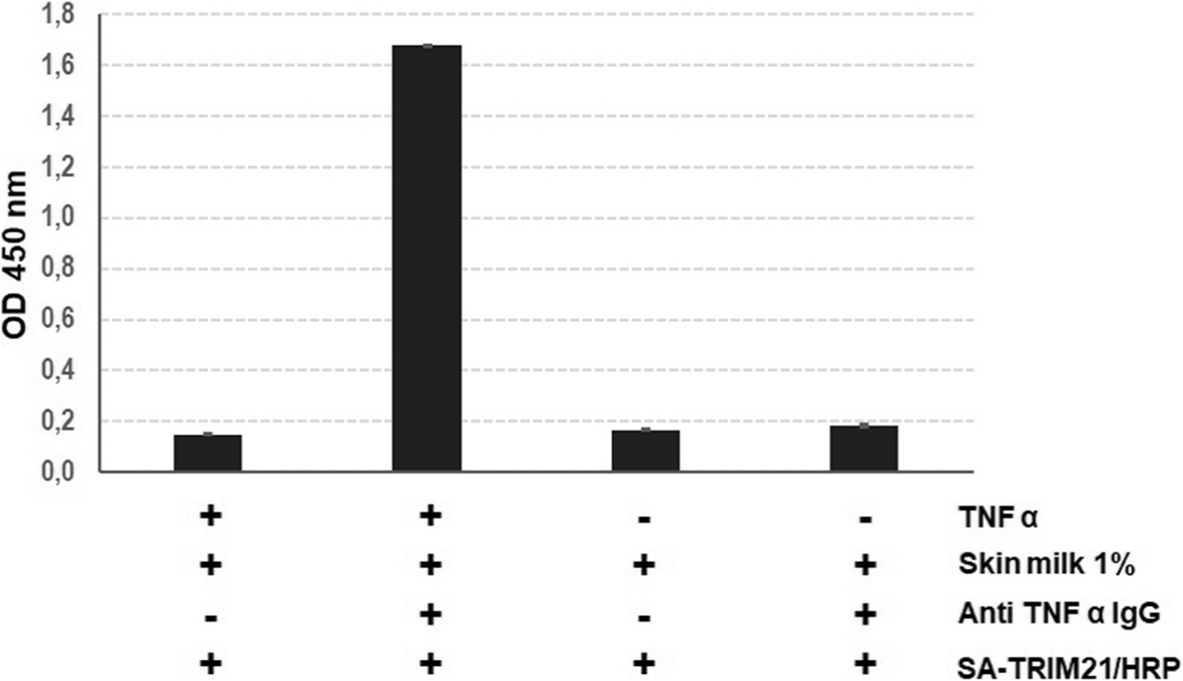
TNF-α/Infliximab detection on indirect ELISA using SA-TRIM21. SA-TRIM21 (0.25 μg/well) and biotinylated HRP (1:5000) were added to the plates for TNF α/Infliximab complex detection

In addition, we tested SA-TRIM21 as a secondary antibody substitute on an indirect ELISA assay (Fig. 3). For that, we coated microtiter plates with 75 ng purified tumor necrosis factor-alpha protein (TNF-α). After, we added diluted human serum containing infliximab (anti-TNF-α IgG1). Figure 3 shows that the chimeric protein efficiently detected infliximab bound to TNF-α.

**Fig. 4:**
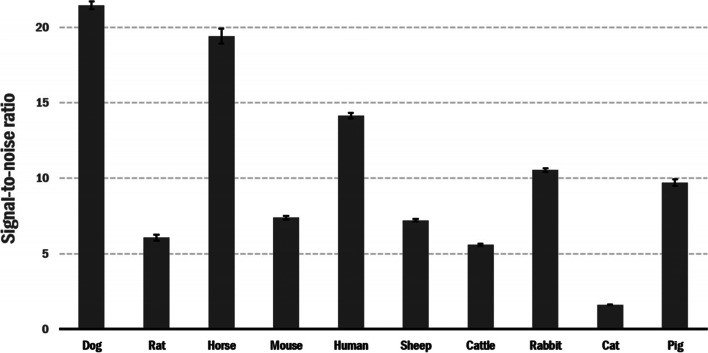
The graph represents the recognition of multispecies sera by the SA-TRIM21-HRP protein. Animal sera (50 ng) were coated onto polystyrene plates, blocked with 1%BSA, incubated with SA-TRIM21 (0.5 μg/well), and biotinylated HRP (1:2500). Control experiments were performed using immobilized BSA into wells. After washing with PBST (low salt), 50 μL chromogenic substrate TMB was added to the wells, incubated for 10 min, followed by the addition of 50 μL H2SO4 to quench the reaction followed by OD_450_ nm reading using an ELISA plate reader. The data shows the ratio of serum/control OD _450_ reading. Experiments were performed in triplicates, and error bars represent standard deviation

Experiments using serum from a wide range of animals were performed to investigate chimeric protein specificity, and data is shown in Fig. 4. As can be observed, SA-TRIM21 recognizes serum antibodies from bovine, mouse, dog, rat, horse, pig, sheep, rabbit, and humans, but not cats.

**Fig. 5:**
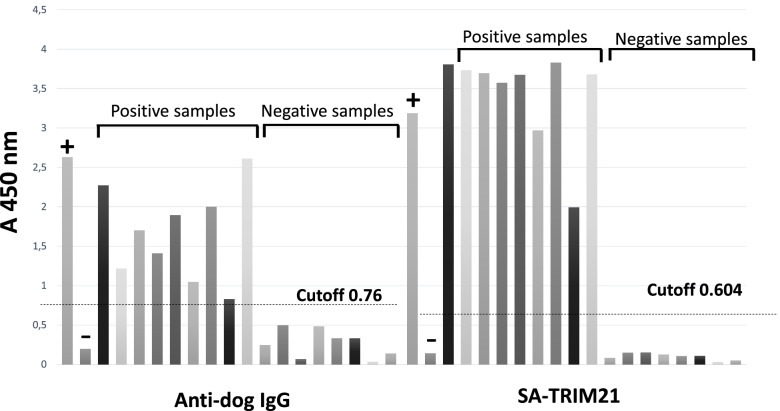
Performance of SA-TRIM21 for IgG detection on veterinary ELISA kits: Anti-Leishmania IgG detection in dog serum. Wells were coated with specific *L. infantum* antigens, followed by incubation with dog serum samples. Wells were washed followed by incubation with HRP coupled anti-IgG or SA-TRIM21. After washing, the chromogen TMB was added to the wells followed by incubation during 10 min. Reactions were quenched using Stop solution and absorbance was read at 450 nm

**Fig. 6:**
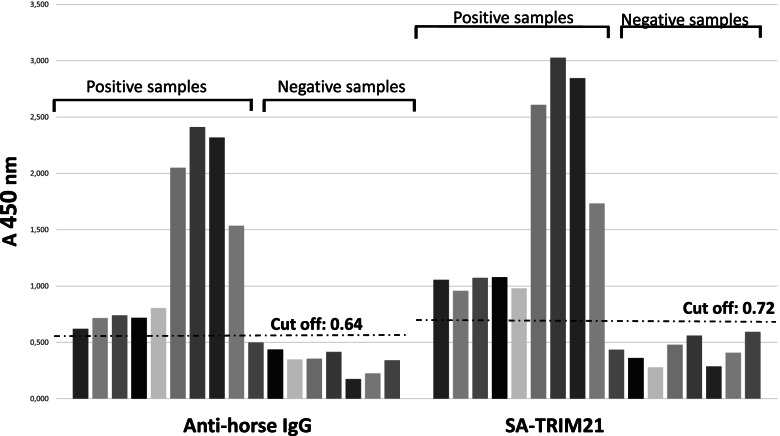
Detection of anti-equine infectious anemia IgG on horse serum. Wells were coated with viral antigens, followed by incubation with horse serum samples. Wells were washed followed by incubation with HRP coupled anti-IgG or SA-TRIM21. After washing, the chromogen TMB was added to the wells followed by incubation during 10 min. Reactions were quenched using Stop solution and absorbance was read at 450 nm

Given the high sensitivity towards dog serum, we tested the performance of SA-TRIM21 for anti-Leishmania IgG detection on dog serum samples using a commercial kit from Bioclin® (Fig. 5). We followed the manufacturer’s protocol and substituted the HRP-coupled anti-IgG for biotin-HRP coupled SA-TRIM21 (0.5 μg/well). Results are presented in Fig. 5. As observed, SA-TRIM21 outperformed polyclonal anti-IgG for this system. We also tested SA-TRIM21 ability to detect horse IgG on an equine infectious anemia ELISA kit. Likewise, we followed the manufacturer’s protocol and compared the performance of SA-TRIM21 (0.25 μg/well) with that of polyclonal anti-horse IgG. Results are shown in Fig. 6. In this case, SA-TRIM21 successfully distinguished negative and positive samples; however, signal/noise ratios of polyclonal anti-horse IgG were marginally superior.

**Fig. 7:**
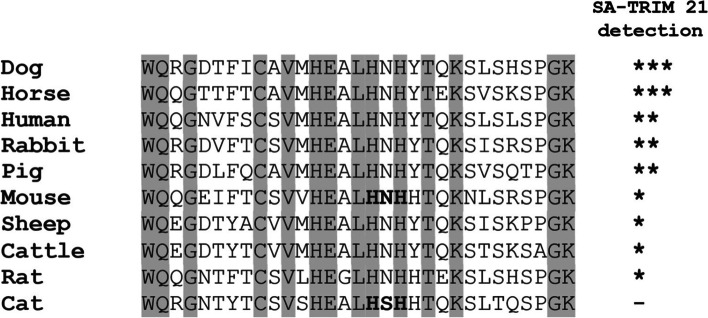
The figure depicts the alignment of IgG heavy chain Fc regions from different mammalian species. The residues in gray are conserved among all sequences. The residues marked in bold represent the Mouse IgG Fc hot spots (H433, N434, and H435) necessary for binding to TRIM21 identified in previous studies [6]

**Fig. 8:**
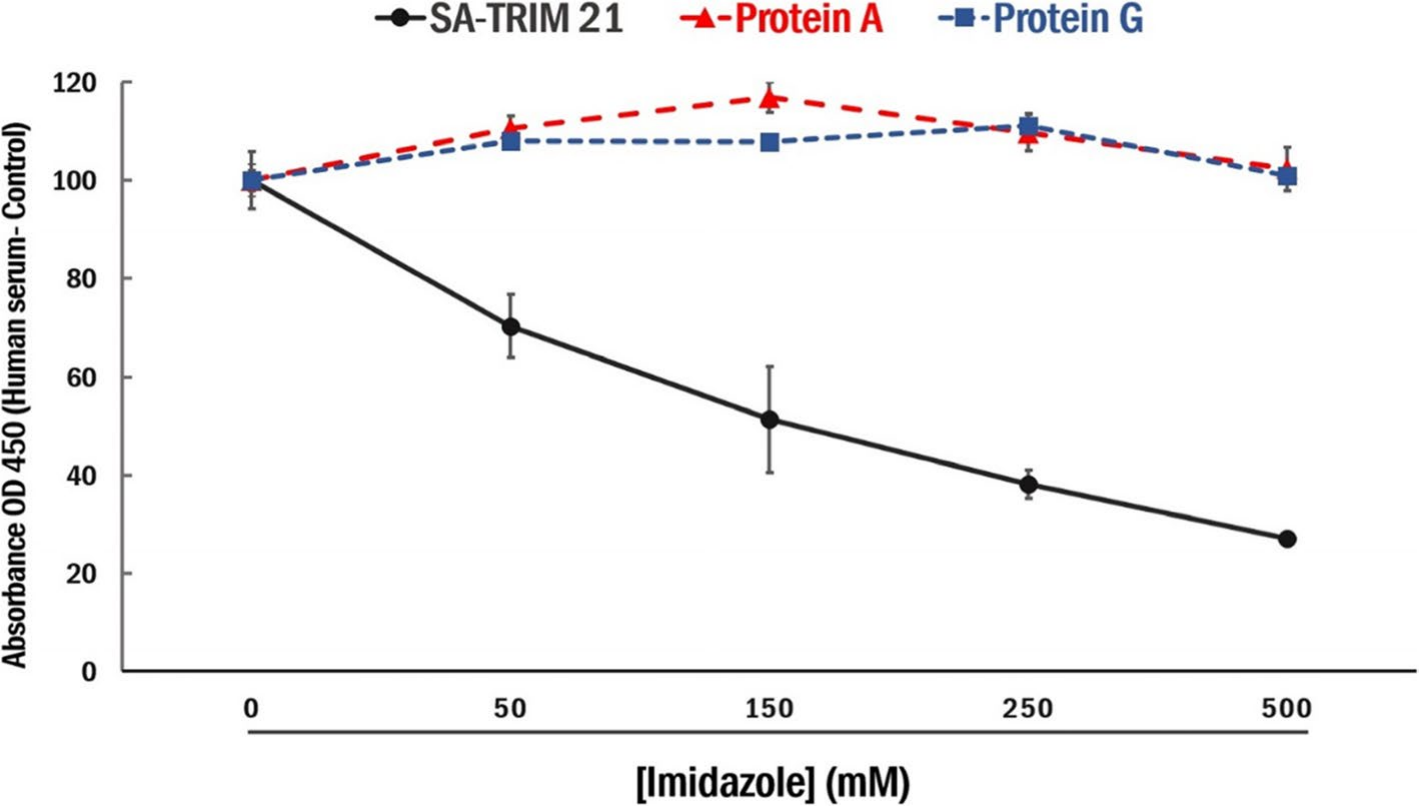
Impact of imidazole elution on IgG binding. Direct ELISA assays using 50 ng immobilized human serum or 50 ng BSA, followed by SA-TRIM21-HRP (0.5 μg) or HRP-coupled Protein A/G incubation and washing with imidazole-containing buffers. Chromogenic substrate TMB was added to the wells, incubated for 10 min, followed by the addition of 50 μL H 2SO4 and OD450 reading. The data shows the ratio of serum/control OD450 reading. Experiments were performed in triplicates, and error bars represent standard deviation

Protein alignment studies demonstrated that the HNH motif is highly conserved among all tested species, except cats (Fig. 7). Therefore, the absence of SA-TRIM21 binding in cat serum indicates that Serine at position 434 disrupts IgG binding. Since two Fc hotspot residues are histidines, we reasoned that imidazole elution might be a potential strategy for IgG elution. Therefore, we performed direct ELISA assays using immobilized horse serum to confirm this hypothesis, followed by washing with imidazole-containing buffers (Fig. 8). One can observe that imidazole disrupts SA-TRIM21-IgG binding but not Protein A/G-IgG binding.

Previous studies demonstrated that TRIM21 binding into IgG is sensitive to high salt concentrations [8]. Therefore, we investigated the role of increasing ionic strength in binding. As shown in Fig. S2, PBST (10 mM NaCl) improved IgG detection compared to regular PBST (137 mM NaCl). Therefore SA-TRIM21 based immunoassays will require reduced ionic strength conditions for binding/detection.

Comparison with protein A and G coupled to HRP from two different vendors using serum from numerous species demonstrated that SA-TRIM21 is a multispecies detection probe with broader specificity than traditional protein A and G detection probes (Fig. S3).

**Fig. 9:**
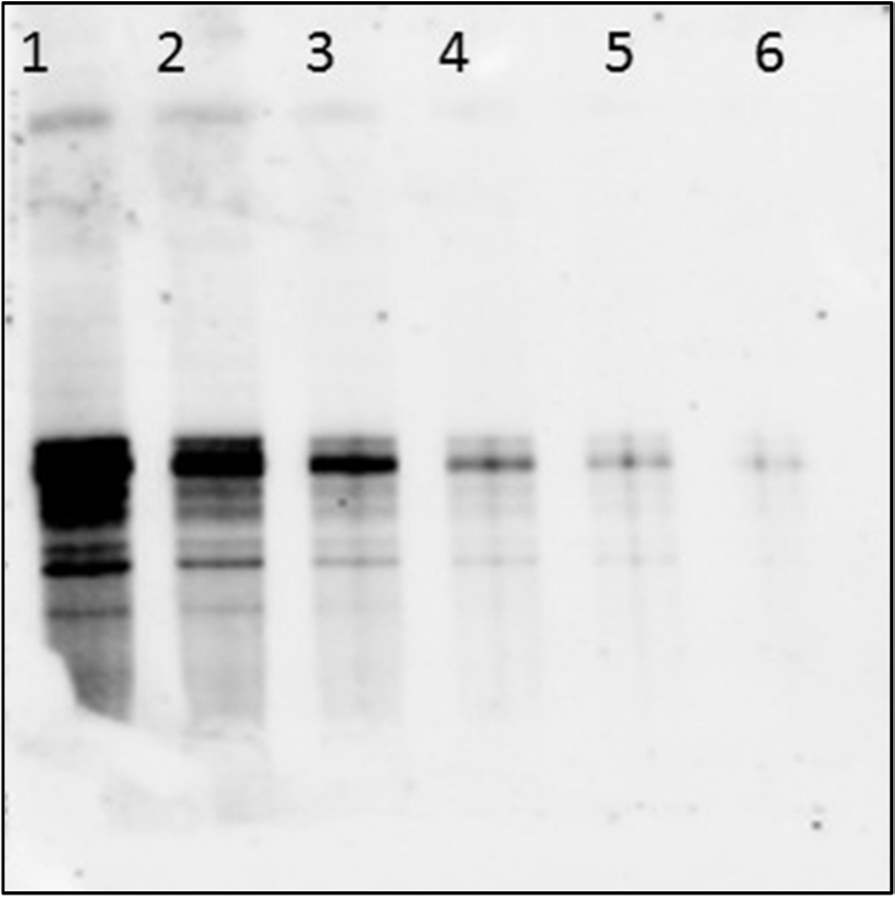
Western blotting detection of CYP1A from protein extracts of mullet fish microsomes. Lane 1, 250 ng of protein extract; lane 2, 125 ng; lane 3, 62.5 ng; lane 4 3.12 ng; lane 5 1.56 ng; lane 6 0.78 ng. Rabbit IgG anti CYP1A was used as the primary antibody at a dilution ratio of 1:7500 in TBS/1% casein. SA-TRIM21 at 0.25 μg/mL was added to the nitrocellulose membrane in TBS/1% casein for rabbit IgG recognition. Biotin HRP (1:2500) was added followed by detection using Luminol based reagent (ECL Scienco Biotech) and chemioluminescent detector

SA-TRIM21 was also used for IgG detection in western blot analysis (Fig. 9), demonstrating that the chimeric protein efficiently recognizes membrane-bound Abs.

## Discussion

In this study, the mouse TRIM21-PRYSPRY S370L mutant was chosen since previous alanine scanning studies demonstrated high enthalpy contributions of this residue (Leucine 370) upon TRIM21 binding into human and murine IgGs [6]. Therefore, a protein with broader IgG specificity was intended by creating a module that detects both human and mouse IgG. The chimeric SA-TRIM21 gene was designed, so the C-terminal PRYSPRY domain binds IgGs, and the streptavidin domain is freely available for biotin binding, therefore not precluding PRYSPRY domain binding into IgG (Fig. 1C).

The EAAAK is a rigid alpha helix-forming linker applied to constructing many recombinant fusion proteins [2]. The α-helical structure with intra-segment hydrogen bonds and a closely packed backbone is a rigid spacer. Therefore this linker was chosen to minimize possible interactions between the two protein domains. In addition, the streptavidin core moiety (residues 13–139) was used because removing streptavidin N and C termini is necessary for the high-affinity biotin-binding to allow coupling of biotinylated enzymes, fluorophores, and nanoparticles [19].

The heterologous expression in *E. coli* remains one of the most attractive protein production strategies, given the bacteria’s fast growth rate at high densities in inexpensive media, enabling an economical production of recombinant proteins [22, 24]. The chimeric protein TRIM21-SA was expressed as inclusion bodies since the streptavidin moiety is toxic for cells. Therefore, expressed proteins were denatured in the presence of Guanidine Hydrochloride followed by simple refolding through dialysis. The amount of protein was estimated by comparing the density of TRIM21-SA against a known mass of BSA from Comassie blue-stained gels. This methodology was necessary since Guanidine Hydrocholide interferes with protein measurements. Insoluble expression resulted in the total production of 100 mg/L of denatured protein, yielding 10 mg/L of soluble and active protein after refolding. The resulting 10% recovery efficiency still represents a feasible choice for large-scale production, considering future efforts on refolding optimization strategies, the straightforward scale-up production, low cost, and good reproducibility of developed protocol.

According to the affinity studies, binding affinity towards the mouse, human and canine IgGs are very similar. Therefore, the increased signal observed for canine and human sera compared to mice might result from S370L mutation or more significant IgG titers on these species.

SA-TRIM21 recognizes serum antibodies from several species except for cats. This data is consistent with previous studies using the C-terminal domain of mouse TRIM21, which demonstrated that interaction is highly conserved, displaying broad cross-species reactivity from a range of mammals [8]. In addition, protein alignment studies demonstrated that the HNH motif is conserved among tested species, except cats, which suggests the importance of Serine 434 for binding of the chimeric protein.

In addition, alanine scanning studies on the residues involved in binding depicted four hot spot residues on TRIM21 (D355, W381, W383, and F450) and three hot spot residues on the mouse Fc domain (H433, N434, and H435) [6]. Therefore, the presence of hotspot histidines involved in binding allowed the assessment of an IgG imidazole elution strategy. Furthermore, one can observe that imidazole disrupts SA-TRIM21-IgG binding but not Protein A/G-IgG binding. Therefore, the possibility of IgG capture using SA-TRIM21 followed by imidazole elution may represent an innovative IgG purification strategy based on neutral pH procedures. Additional studies to assess the potential of the chimeric protein on SA-TRIM21-based resins for IgG purification are currently underway.

The interaction between the C terminal PRYSPRY domain in complex with mouse IgG1 has been previously solved, allowing a deep understanding of the molecular interactions of the complex [6]. Analysis of the IgG-PRYSPRY complex revealed binding of one PRYSPRY domain on each Fc side, resulting in a 2:1 stoichiometry. The full-length protein dimerizes through its coiled-coil domain. Upon IgG binding, it engages both heavy chains simultaneously, resulting in higher affinity constants (K_d_ = 0.5 nM) than the monomeric C-terminal PRYSPRY domain [6].

Since bacterial proteins A and G have complementary binding patterns, a fusion protein enclosing both proteins generated broader reporter molecules [3, 21]. More recently, engineered proteins were selected to recognize different epitopes within human IgG and exhibited binding affinities in the nanomolar range [1]. However, although IgG binders of high thermal and pH stability were generated, they mostly recognized human IgGs of subclasses 1 and 2, therefore hampering their use as multispecies molecular tools for IgG detection.

Data reported here demonstrates that SA-TRIM21 chimeric protein is an efficient and multispecies IgG detection probe for ELISA and western blot analysis. The development of new Ab-affinity ligands (recombinantly expressed in *E. coli*) is crucial to assist IgG detection and pave the way for new purification strategies. The efficient elution using imidazole is an important feature that may allow the development of IgG affinity resins using neutral pH and standard imidazole-containing buffers.

## Methods

### Materials

The following IgG detection probes were used for immunoassays: HRP-conjugated Protein A (#95217)/ HRP-conjugated Protein G (#64256837) were purchased from Bio-Rad® (Hercules, CA, USA). HRP-conjugated Protein A (#101023)/HRP-conjugated Protein G (#101223) were purchased from Thermo-Fisher (Waltham, MA USA). HRP-conjugated anti-mouse IgG (#A4416, Sigma), HRP-conjugated anti-cattle IgG (#A5295, Sigma), HRP-conjugated anti-dog IgG (#A6792, Sigma), HRP-conjugated anti-rat IgG (#A9037, Sigma), HRP-conjugated anti-horse (#A6917, Sigma), HRP-conjugated anti-pig IgG (#A5670, Sigma) and HRP-conjugated anti-human IgG (#A170, Sigma) were used in this study. ELISA kits for visceral leishmaniosis (REF: #VET043-1) and equine infectious anemia (REF: #VET037-1) were kindly provided by Bioclin®. Scienco Biotech kindly provided tetramethylbenzidine (ONE STEP-TMB Linear, Scienco Biotech).

### Chimeric protein gene design

Streptavidin-TRIM21 (SA-TRIM21) chimeric gene was designed by insertion of the wild-type core streptavidin coding sequence (residues 13–140 of the mature full-length protein) [19] at the N-terminal end of the construct, followed by a rigid linker (five repetitive sequences of EAAAK) and the C-terminal PRYSPRY domain (S370L) gene sequence of mouse TRIM21. SA-TRIM21 gene sequence was synthesized and cloned into pET15b(+) vector between *Nde* I e *Xho* I restriction sites and purchased from Genescript ®. Their schematic design of the primary structure and the putative quaternary structure is shown in Fig. 1B.

### Protein expression and purification

Recombinant plasmids were transformed into *E. coli* BL21 pLysS (DE3). Cells were inoculated into Luria-Bertani Broth (LB) media containing appropriate antibiotics (ampicillin 100 μg/mL and chloramphenicol 35 μg/mL) and biotin 2 μg/mL and cultivated at 37 ^o^C until reach 600 nm optical density (OD) of 0.6 followed by induction with 0.1 mM IPTG at 37 °C for 12 h. Cells were centrifuged, and the bacterial pellet was suspended in 50 mM Tris, 300 mM NaCl containing 50 mg DNASE, 100 mg/mL PMSF, and 1 mg/mL lysozyme followed by cell disruption through sonication. The bacterial cell lysate was centrifuged, and the insoluble cell fraction was washed five times with 50 mM Tris pH 8.0, 300 mM NaCl, 5 mM EDTA, 0.1% Triton X-100 and centrifuged at 10,000 rpm for 10 min. The insoluble pellet was then solubilized with 6 M guanidine hydrochloride, 20 mM phosphate pH 1.5 for 1h at 37 °C and dialyzed against Refolding Buffer (0.2 M ammonium acetate, 0.02% sodium azide, 5% sucrose, PMSF and 0.1% Tween-20) for protein refolding. The refolded protein extract was then dialyzed against 50 mM Tris pH 8.0, 300 mM NaCl, 0.1% Tween-20, and applied onto a Ni-NTA Resin. The resin was washed with 10 column volumes (CV) of 50 mM Tris-HCl pH 8.0, 40 mM imidazole to remove weakly bound proteins, and protein was eluted with 3.5 CV of 50 mM Tris-HCl pH 8, 300 mM imidazole.

### Western blot analysis

Protein extracts were fractionated by SDS-PAGE and transferred into a nitrocellulose membrane according to the manufacturer's protocols (Bio-Rad®). After incubation with 1% skimmed milk in TBST for 2 h, the membrane was washed three times with TBST and incubated with mouse anti-His-tag antibody (Sigma-Aldrich®, 1:5000), at room temperature for 2 h. For HRP labeled western blots, membranes were washed three times with TBST-Low salt (10 mM NaCl) and incubated with 2.5 mg/mL HRP conjugated SA-TRIM21 or HRP-conjugated anti-mouse IgG for 2 h. Blots were washed with TBST-Low salt (10 mM NaCl) three times and developed with ECL-Luminol (Scienco Biotech) according to the manufacturer’s protocols.

### Enzyme-linked immunosorbent assay (ELISA) analysis

All ELISA assays were performed into 96 wells high binding microtiter plates. For direct ELISA assays, wells were coated (18 h, 4 °C) with purified IgG or animal serum in 50 μL coating buffer (0.05 M Na_2_CO3, 0.05 M NaHCO_3_, pH 9.6) and blocked with 1% BSA in TBST. Next, HRP coupled SA-TRIM21 was added to the wells and incubated for 1 h at room temperature. Plates were washed five times with PBST-Low salt (10 mM NaCl) after each step. The reaction was visualized by adding 50 μL chromogenic substrate (ONE STEP TMB, Scienco Biotech) for 10 min. The reaction was quenched with 50 μL H_2_SO_4_ 0.16 N, and absorbance at 450 nm was measured using an ELISA plate reader.

Indirect Elisa assays were performed immobilizing TNF alpha on high binding microtiter plates in 50 μL coating buffer (0.05 M Na_2_CO_3_, 0.05 M NaHCO_3_, pH 9.6) and blocked with 1% BSA. Next, anti-TNF-alpha IgG (3 μg/mL) was added to the wells for 1h at room temperature. Plates were washed five times with TBST and added SA-TRIM-HRP (2.5 μg/mL) or anti-human HRP IgG (Sigma-Aldrich®) to the wells (1 h, room temperature). The reaction was visualized by the addition of 50 μL chromogenic substrate (TMB) for 30 min. The reaction was stopped with 50 μL H_2_SO_4_ and absorbance at 450 nm was measured using an ELISA plate reader.

ELISA assays for visceral leishmaniosis and equine infectious anemia (EIA) were performed using commercial kits. According to the manufacturer's information, the cut-offs of both products were determined by evaluating a panel of samples previously characterized by a reference serological method. In addition, obtained results were evaluated by receiver operating characteristic (ROC) analysis. According to ROC analysis, cut-offs were calculated using Eqs. 1 and 2, as follows:

1 $${\displaystyle \begin{array}{l}\mathrm{Cut}-\mathrm{off}\ \mathrm{visceral}\ \mathrm{leishmaniosis}:\left(\mathrm{Average}\ \mathrm{absorbance}\ \mathrm{negative}\ \mathrm{sample}\right)+0.5\\ {}\mathrm{Leishmaniosis}\ \mathrm{cut}-\mathrm{off}\ \left(\mathrm{anti}-\mathrm{dog}\ \mathrm{IgG}\ \mathrm{antibody}\right):\mathrm{Negative}\ \mathrm{sample}\ \mathrm{average}\ (0.269)+0.5=0.769\\ {}\mathrm{Leishmaniosis}\ \mathrm{cut}-\mathrm{off}\ \left(\mathrm{SA}-\mathrm{TRIM}21\right):\mathrm{Negative}\ \mathrm{sample}\ \mathrm{average}\ (0.104)+0.5=0.604\end{array}}$$

and

2 $${\displaystyle \begin{array}{l}\mathrm{Cut}-\mathrm{off}\ \mathrm{equine}\ \mathrm{infectious}\ \mathrm{anemia}:\left(\mathrm{Average}\ \mathrm{absorbance}\ \mathrm{negative}\ \mathrm{sample}\right)+0.3\\ {}\mathrm{EIA}\ \mathrm{test}\ \left(\mathrm{anti}-\mathrm{horse}\ \mathrm{IgG}\ \mathrm{antibody}\right):\mathrm{Negative}\ \mathrm{sample}\ \mathrm{average}\ (0.349)+0.3=0.649\\ {}\mathrm{EIA}\ \mathrm{test}\ \left(\mathrm{SA}-\mathrm{TRIM}21\right):\mathrm{Negative}\ \mathrm{sample}\ \mathrm{average}\ (0.425)+0.3=0.725\end{array}}$$

## Conclusion

Secondary antibody production methods have recently relied on recurrent animal immunization and serum harvesting and purification. These batches, enriched with anti-IgG polyclonal antibodies; continue to be used as high-quality reagents for research and diagnostics. However, the inherent technical limitations and ethical concerns raised by polyclonal antibodies have prompted scientists to develop animal-free techniques for secondary antibody production. More importantly, mAbs for diagnostic and therapeutic use has increased extensively, currently imposing the need for an improved portfolio of affinity ligands for IgG detection and purification. Data reported here provides an additional and superior alternative to the use of secondary antibodies, expanding the portfolio of antibodies affinity ligands.

## Supplementary Information

**Additional file 1: Figure S1.** The best stoichiometric ratios between SA-TRIM21 and biotinylated HRP.

**Additional file 2: Figure S2.** PBST (10 mM NaCl) improved IgG detection compared to regular PBST.

**Additional file 3: Figure S3.** Comparison with protein A and G coupled to HRP from two different vendors using serum from numerous species demonstrated that SA-TRIM21 is a multispecies detection probe with broader specificity than traditional protein A and G detection probes.

## Data Availability

The datasets used and/or analyzed during the current study are available from the corresponding author on reasonable request.

## Abbreviations

Abs: Antibodies
TRIM21: Tripartite motif-containing protein 21
IgG: G-type immunoglobulins
ELISA: Enzyme-linked immunoassay
SA: Streptavidin
