# Supplementary figures and images for: TRIM21 chimeric protein as a new molecular tool for multispecies IgG detection
Source: J Genet Eng Biotechnol. 2022 Jul 28;20:111. doi: 10.1186/s43141-022-00396-3 (PMC9334459; doi:10.1186/s43141-022-00396-3)

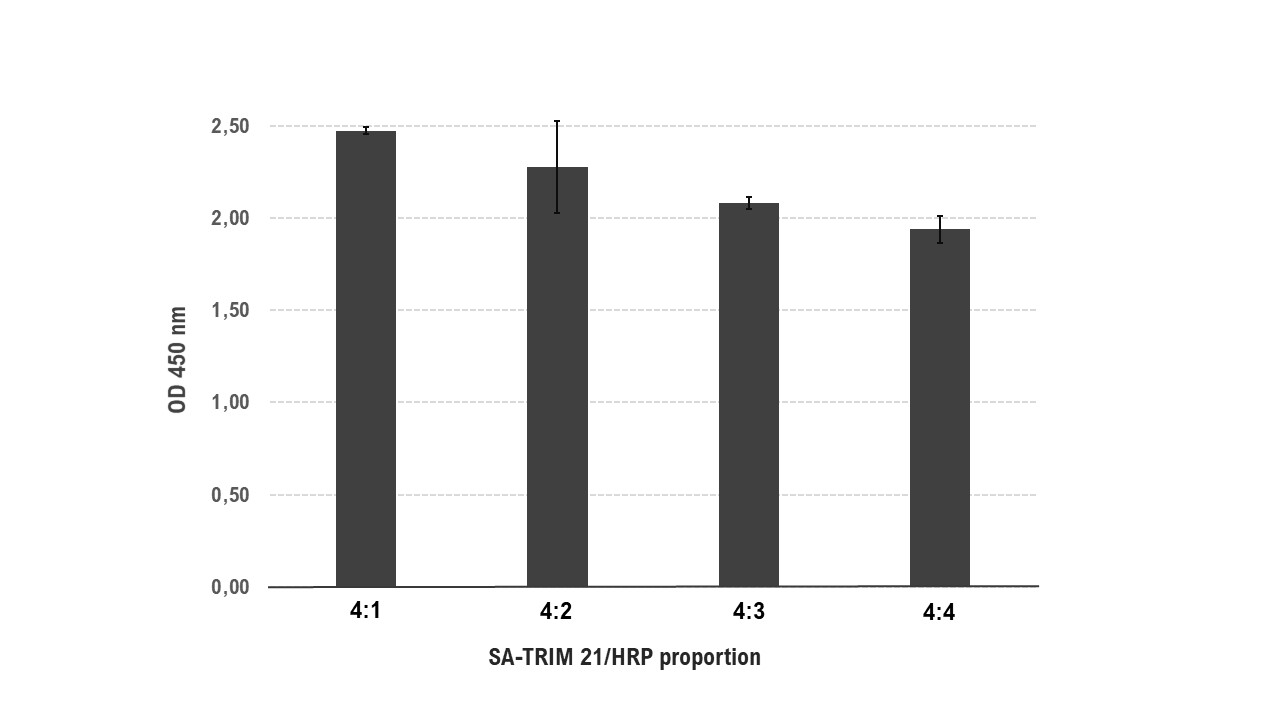

Supplement: Supplementary file 1 — Additional file 1: Figure S1. The best stoichiometric ratios between SA-TRIM21 and biotinylated HRP. [file 43141_2022_396_MOESM1_ESM.jpg]

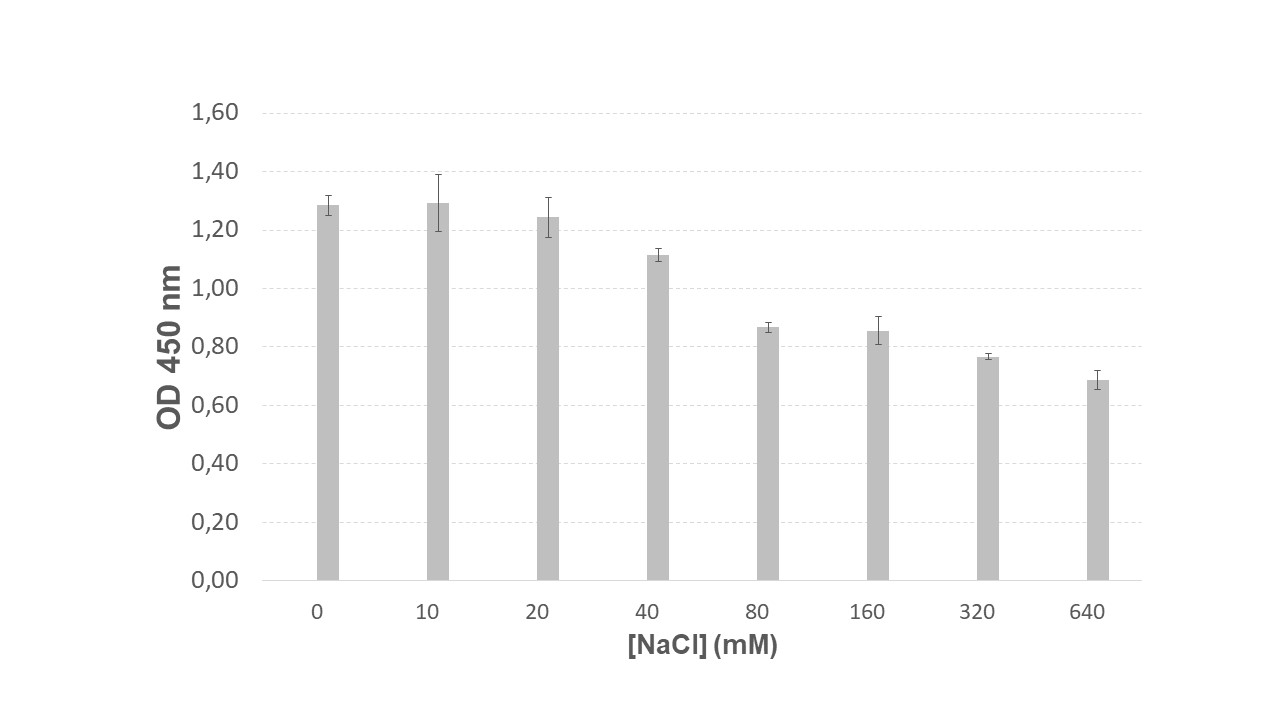

Supplement: Supplementary file 2 — Additional file 2: Figure S2. PBST (10 mM NaCl) improved IgG detection compared to regular PBST. [file 43141_2022_396_MOESM2_ESM.jpg]

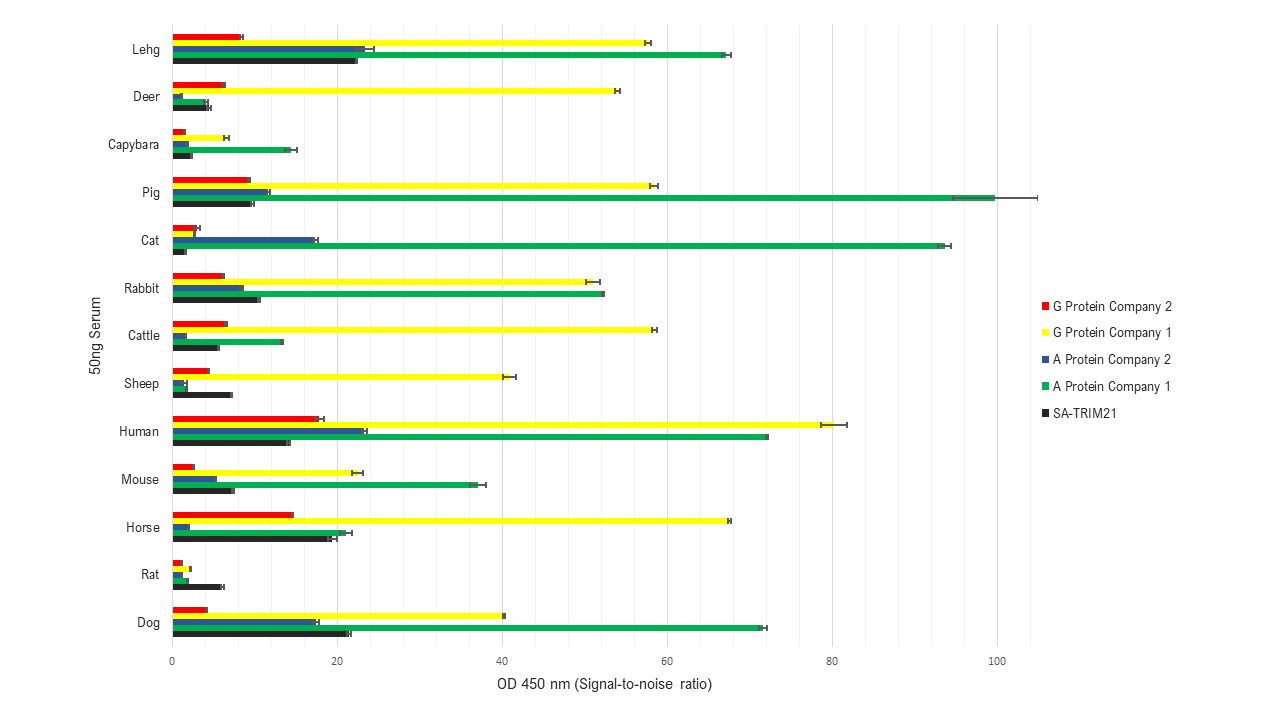

Supplement: Supplementary file 3 — Additional file 3: Figure S3. Comparison with protein A and G coupled to HRP from two different vendors using serum from numerous species demonstrated that SA-TRIM21 is a multispecies detection probe with broader specificity than traditional protein A and G detection probes. [file 43141_2022_396_MOESM3_ESM.jpg]
